# Low-Level Environmental Lead Exposure and Children’s Intellectual Function: An International Pooled Analysis

**DOI:** 10.1289/ehp.7688

**Published:** 2005-03-18

**Authors:** Bruce P. Lanphear, Richard Hornung, Jane Khoury, Kimberly Yolton, Peter Baghurst, David C. Bellinger, Richard L. Canfield, Kim N. Dietrich, Robert Bornschein, Tom Greene, Stephen J. Rothenberg, Herbert L. Needleman, Lourdes Schnaas, Gail Wasserman, Joseph Graziano, Russell Roberts

**Affiliations:** ^1^Cincinnati Children’s Hospital Medical Center, Cincinnati, Ohio, USA; ^2^Department of Environmental Health, University of Cincinnati College of Medicine, Cincinnati, Ohio, USA; ^3^Institute for Health Policy and Health Services Research, Department of Environmental Health, University of Cincinnati, Cincinnati, Ohio, USA; ^4^Women and Children’s Hospital, North Adelaide, South Australia; ^5^Department of Neurology, Children’s Hospital Boston and Harvard Medical School, Boston, Massachusetts, USA; ^6^Division of Nutritional Sciences, Cornell University, Ithaca, New York, USA; ^7^Department of Biostatistics and Epidemiology, Cleveland Clinic Foundation, Cleveland, Ohio, USA; ^8^Center for Research in Population Health, National Institute of Public Health, Cuernavaca, Morelos, Mexico; ^9^Drew University, Los Angeles, California, USA; ^10^University of Pittsburgh School of Medicine, Pittsburgh, Pennsylvania, USA; ^11^National Institute of Perinatology, Mexico City, Mexico; ^12^Department of Child Psychiatry, Columbia University, New York, New York, USA; ^13^Department of Environmental Health Sciences, Columbia University, New York, New York, USA; ^14^School of Applied Psychology, Griffith University, Queensland, Australia

**Keywords:** blood lead concentration, children, environment, epidemiology, intelligence, lead, lead toxicity

## Abstract

Lead is a confirmed neurotoxin, but questions remain about lead-associated intellectual deficits at blood lead levels < 10 μg/dL and whether lower exposures are, for a given change in exposure, associated with greater deficits. The objective of this study was to examine the association of intelligence test scores and blood lead concentration, especially for children who had maximal measured blood lead levels < 10 μg/dL. We examined data collected from 1,333 children who participated in seven international population-based longitudinal cohort studies, followed from birth or infancy until 5–10 years of age. The full-scale IQ score was the primary outcome measure. The geometric mean blood lead concentration of the children peaked at 17.8 μg/dL and declined to 9.4 μg/dL by 5–7 years of age; 244 (18%) children had a maximal blood lead concentration < 10 μg/dL, and 103 (8%) had a maximal blood lead concentration < 7.5 μg/dL. After adjustment for covariates, we found an inverse relationship between blood lead concentration and IQ score. Using a log-linear model, we found a 6.9 IQ point decrement [95% confidence interval (CI), 4.2–9.4] associated with an increase in concurrent blood lead levels from 2.4 to 30 μg/dL. The estimated IQ point decrements associated with an increase in blood lead from 2.4 to 10 μg/dL, 10 to 20 μg/dL, and 20 to 30 μg/dL were 3.9 (95% CI, 2.4–5.3), 1.9 (95% CI, 1.2–2.6), and 1.1 (95% CI, 0.7–1.5), respectively. For a given increase in blood lead, the lead-associated intellectual decrement for children with a maximal blood lead level < 7.5 μg/dL was significantly greater than that observed for those with a maximal blood lead level ≥7.5 μg/dL (*p* = 0.015). We conclude that environmental lead exposure in children who have maximal blood lead levels < 7.5 μg/dL is associated with intellectual deficits.

The preponderance of experimental and human data indicates that there are persistent and deleterious effects of blood lead levels > 10 μg/dL on brain function, including lowered intelligence, behavioral problems, and diminished school performance (Baghurst et al. 1992; Bellinger et al. 1992; Cory-Slechta 1997; Dietrich et al. 1993; Ernhart et al. 1989; National Research Council 1993; Needleman and Gatsonis 1990; Pocock et al. 1994; Rice 1993; Wasserman et al. 1997; Yule et al. 1981). Lead toxicity, defined as whole blood lead ≥10 μg/dL, was based on numerous cross-sectional and prospective studies [Bellinger et al. 1987; Centers for Disease Control and Prevention (CDC) 1991; World Health Organization (WHO) 1995]. These studies generally, but not always, found adverse consequences of childhood lead exposure (CDC 1991; WHO 1995). Still, most of the children in those studies had blood lead levels > 10 μg/dL. The WHO and the CDC recognized that there was no discernable threshold for the adverse effects of lead exposure, but too few studies had examined children with blood lead levels < 10 μg/dL to support any firm conclusions (CDC 1991; WHO 1995).

There is emerging evidence that lead-associated intellectual deficits occur at blood lead levels < 10 μg/dL. In the Rochester Longitudinal Study, there was an estimated reduction of 7.4 IQ points associated with an increase in lifetime mean blood lead from 1 to 10 μg/dL (Canfield et al. 2003). In a reanalysis of a Boston, Massachusetts, cohort, a similar finding was observed among children whose maximal blood lead level was < 10 μg/dL (Bellinger and Needleman 2003). Questions about an effect of lead at blood lead levels < 10 μg/dL persist, however, because of the relatively small numbers of children with maximal blood lead levels < 10 μg/dL in the Rochester Longitudinal Study (Rogan and Ware 2003). Other studies were limited because they involved children whose blood lead levels may have exceeded 10 μg/dL at some point in their lifetime or because important covariates, such as maternal IQ scores, were not always available (Fulton et al. 1987; Lanphear et al. 2000; Schwartz 1994; Schwartz and Otto 1991; Walkowiak et al. 1998). Because of the policy implications of this research, it is critical to estimate with greater precision the exposure–response relationship at blood lead levels < 10 μg/dL.

The primary objective of this pooled analysis was to estimate the quantitative relationship between children’s performance on IQ tests and selected measures of blood lead concentration among children followed prospectively, from infancy through 5–10 years of age in seven prospective cohort studies. We also sought to test whether the lead-associated IQ deficit was greater for a given change in exposure among children who had maximal blood lead levels < 10 μg/dL compared with children who had higher blood lead concentrations.

## Materials and Methods

We contacted investigators for all eight prospective lead cohorts that were initiated before 1995, and we were able to retrieve data sets and collaboration from seven. The participating sites were Boston (Bellinger et al. 1992); Cincinnati (Dietrich et al. 1993) and Cleveland, Ohio (Ernhart et al. 1989); Mexico City, Mexico (Schnaas et al. 2000); Port Pirie, Australia (Baghurst et al. 1992); Rochester, New York (Canfield et al. 2003); and Yugoslavia (Wasserman et al. 1997). The Sydney, Australia, study was not included because we were unable to contact the investigators (Cooney et al. 1989). The data for the Rochester Longitudinal Study and for Mexico City, collected when the children were about 6 years of age, have not been published elsewhere. The eligibility criteria and methods for each of the cohorts are described elsewhere (Baghurst et al. 1992; Bellinger et al. 1992; Canfield et al. 2003; Dietrich et al. 1993; Ernhart et al. 1989; Schnaas et al. 2000; Wasserman et al. 1997). All studies were approved by their respective institutional review boards.

### Outcome measures.

The primary outcome measure was the full-scale IQ, which is a composite score of verbal and performance tests. The children were administered a version of the Wechsler Intelligence Scales for Children [Wechsler Intelligence Scale for Children–Revised (WISC-R; Wechsler 1974), Wechsler Intelligence Scale for Children–III (WISC-III; Wechsler 1991), Wechsler Preschool and Primary Scales of Intelligence (WPPSI; 1967), and Wechsler Intelligence Scale for Children–Spanish Version (WISC-S; Wechsler 1981)] under uniform conditions within each study. The IQ test was administered when the children were between 4 years 10 months and 7 years of age for all but one cohort. In the Boston cohort, we used blood lead tests taken at 5 years of age and the nearest available full-scale IQ score, which was done at 10 years of age.

Venous or fingerstick capillary blood samples were obtained using standard protocols. Cord blood lead was collected in a subsample of the subjects. During each child’s examination, demographic and health information were obtained from the parent (usually the biologic mother). IQ tests were administered to the mother. We also obtained data on other factors that might confound the relation of lead exposure and IQ, including child’s sex, birth order, birth weight, maternal education, maternal age, marital status, prenatal alcohol exposure, prenatal tobacco exposure, and the Home Observation for Measurement of the Environment (HOME) Inventory score. The HOME Inventory is an index that reflects the quality and quantity of emotional and cognitive stimulation in the home environment (Caldwell and Bradley 1984).

### Measures of exposure.

We examined four measures of blood lead: concurrent blood lead (defined as the blood lead measured closest to the IQ test), maximum blood lead level (defined as the peak blood lead measured at any time before IQ test), average lifetime blood lead (defined as the mean blood lead from 6 months to concurrent blood lead tests), and early childhood blood lead (defined as the mean blood lead from 6 to 24 months). The blood sampling intervals varied across studies. To enhance comparability across studies, we used the following blood sampling intervals (based on children’s age): 6, 12 (or 15), 36, 48, and 60 months. We used mean blood lead rather than area under the curve (AUC) to maintain the same units of analysis for all four lead indices. The AUC and mean provided essentially the same information about children’s lead exposure (*r* = 0.97).

### Statistical methods.

To estimate the quantitative relationship between children’s performance on IQ tests and selected measures of blood lead concentration, we examined the potential confounding effects of other factors associated with IQ scores using multiple regression analysis. Ten factors were available from individual sites: HOME Inventory, child’s sex, birth weight, birth order, maternal education, maternal IQ, maternal age, marital status, prenatal smoking status, and prenatal alcohol use.

The development of the regression model involved a multistep process beginning with a simple unadjusted model relating each blood lead measure to IQ while controlling for site. The first step was to test whether the linear model of the relationship between blood lead and IQ, applied in most of the individual cohort analyses, provided a good fit over the wider range of blood lead levels represented in the pooled data. First, a linear model adjusted for the seven sites was estimated, and then quadratic and cubic terms for blood lead were added to test for linearity. A restricted cubic spline function was fit to the data to produce a curve that followed the data in the absence of any assumptions about the functional form of the relationship.

After an initial model was chosen, we examined each of the 10 available confounders individually and in combination with the other covariates to assess potential confounding of the IQ–blood lead relationship. Careful attention was paid to the stability of the parameter estimates as each additional term was added. This process was halted when either no more significant terms (*p* < 0.10) entered the model or the inclusion of additional terms caused no substantial change (i.e., > 10%) in the blood lead coefficient.

In all models, we tested the interaction of blood lead and site to determine whether a summary measure of the IQ–blood lead relationship could be used for all cohorts. After an initial model was selected, the tests of linearity and the restricted cubic spline models were recomputed to ensure that our initial model was still appropriate after adjustment for covariates (Harrell 2001). We also produced separate linear models for each of the seven cohorts adjusted for the covariates selected in the combined model.

After the multiple regression models were developed, regression diagnostics were employed to ascertain whether the lead coefficient was affected by collinearity or influential observations (Belsley et al. 1980). After regression diagnostics were examined and homogeneity of the blood lead coefficients across sites was evaluated, the fit of all four measures of blood lead was compared using the magnitude of the model *R*^2^. The blood lead measure with the largest *R* ^2^ (adjusted for the same covariates) was selected *a priori* as the preferred blood lead index relating blood lead to IQ.

Several approaches were investigated to evaluate the stability of the final model. Although the seven cohorts were not randomly sampled from a larger population of studies, an assumption could be made that they were representative of a larger population of children. Accordingly, we evaluated the results of applying a random-effects model (with sites random) rather than a fixed-effects model (Littell et al. 1996). We also examined the effect of any one site on the overall model by calculating the blood lead coefficient in seven identical models, each omitting one of the seven cohorts (Efron and Tibshirani 1993).

After the final model was selected using the full-scale IQ as the outcome variable, we fit similar models for verbal and performance IQ scores. We also examined interactions of covariates with blood lead concentration (effect modification) and tested the effect of including race as a confounder in the U.S. cohort studies. Finally, we examined the relationship of prenatal lead exposure (cord blood) and IQ score in the subsample for which cord blood lead tests were available.

## Results

Of the 1,581 eligible children from the seven cohorts, data on all 10 covariates were available for 1,308 (83%) children; 1,333 (84%) children had data on the four major covariates that were selected for the final model (Table 1). Blood lead levels were highest in Yugoslavia and lowest in Rochester and Boston for all lead exposure indices (Table 2). The median peak or maximal blood lead concentration was 18 μg/dL; the mean age when children’s blood lead levels peaked was 2.5 years. By 5–7 years of age, the median blood level had declined to 9.7 μg/dL (concurrent blood lead concentration). The lifetime average blood lead concentration was 12.4 μg/dL; 244 (18%) children had a maximal blood lead concentration < 10 μg/dL, and 103 (8%) had a maximal blood lead concentration < 7.5 μg/dL.

The mean IQ of all children was approximately 93. Child IQ was highest in the Boston cohort and lowest in the Yugoslavia cohort (Table 2). In univariate regression analyses, children’s IQ was significantly related to site, maternal IQ, the HOME score, maternal education, marital status, birth weight, maternal age, birth order, race (for U.S. cohorts only), and prenatal tobacco exposure. In contrast, child’s sex and prenatal alcohol consumption were not significantly associated with a deficit in IQ score (Table 3).

We examined the relationship of the four blood lead indices with IQ (Table 4). Although all four blood lead measures were highly correlated (*r* range = 0.74–0.96), the concurrent blood lead variable exhibited the strongest relationship with IQ, as measured by *R* ^2^. Although the means differed for the different blood lead indices, the results of the regression analyses were very similar. In all subsequent analyses and figures, the concurrent blood lead measure was used as the primary lead exposure index.

**Figure 1:**
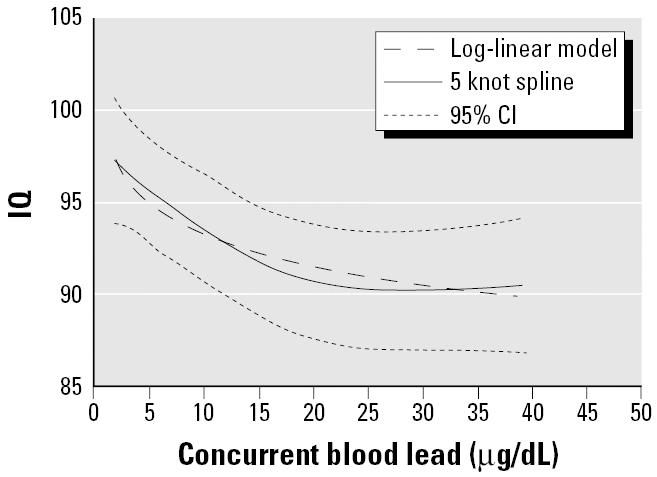
Restricted cubic splines and log-linear model for concurrent blood lead concentration. The dotted lines are the 95% CIs for the restricted cubic splines.

The shape of the exposure–response relationship was determined to be nonlinear insofar as the quadratic and cubic terms for concurrent blood lead were statistically significant (*p* < 0.001 and *p* = and 0.003, respectively). Because the restrictive cubic spline indicated that a log-linear model provided a good fit to the data, we used the log of concurrent blood lead in all subsequent analyses of the pooled data (Figure 1).

**Figure 2:**
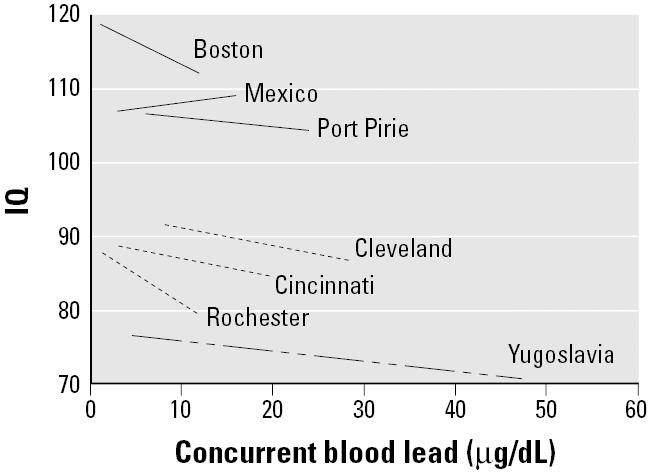
Linear models for each cohort study in the pooled analysis, adjusted for maternal IQ, HOME score, maternal education, and birth weight. The figure represents the 5th to 95th percentile of the concurrent blood lead level at the time of IQ testing.

The multivariable analysis resulted in a six-term model: log of concurrent blood lead, site, maternal IQ, HOME Inventory, birth weight, and maternal education, which we consider our preferred model (Table 4). Linear models of concurrent blood lead and IQ are shown for each of the seven cohorts, adjusted for the same covariates (Figure 2). The additional six terms we considered (child’s sex, birth order, maternal age, marital status, prenatal smoking status, and prenatal alcohol use) contributed very little to the overall fit of the model, and their inclusion in the model resulted in virtually no change to the coefficient for blood lead (i.e., < 5%). None of the six terms was statistically significant (data not shown).

**Figure 3:**
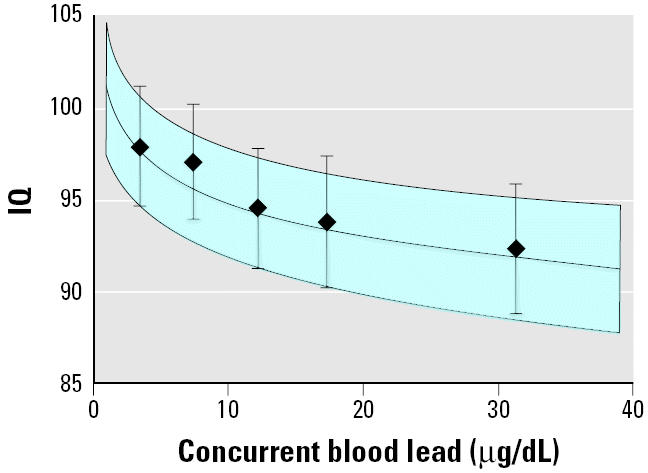
Log-linear model (95% CIs shaded) for concurrent blood lead concentration, adjusted for HOME score, maternal education, maternal IQ, and birth weight. The mean IQ (95% CI) for the intervals < 5 μg/dL, 5–10 μg/dL, 10–15 μg/dL, 15–20 μg/dL, and > 20 μg/dL are shown.

**Figure 4:**
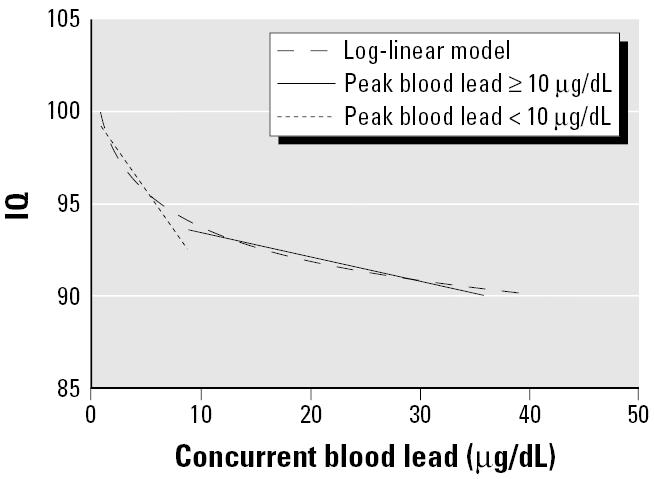
Log-linear model for concurrent blood lead concentration along with linear models for concurrent blood lead levels among children with peak blood lead levels above and below 10 μg/dL.

The shape of the log-linear model and the spline function indicated that the steepest declines in IQ were at blood lead levels < 10 μg/dL (Figures 3 and 4). The log-linear model estimated a decrement of 6.9 IQ points [95% confidence interval (CI), 4.2–9.4] for an increase in concurrent blood lead levels from 2.4 to 30 μg/dL, representing the 5th to the 95th percentile for blood lead values in the data set (Table 4). But the lead-associated decrement was greatest in the lower ranges of blood lead. The estimated IQ decrements associated with an increase in blood lead from 2.4 to 10 μg/dL, 10 to 20 μg/dL, and 20 to 30 μg/dL were 3.9 (95% CI, 2.4–5.3), 1.9 (95% CI, 1.2–2.6), and 1.1 (95% CI, 0.7–1.5), respectively (Table 4).

To investigate further whether the lead-associated decrement was greater at lower blood lead concentrations, we divided the data at two cut-points *a priori* (i.e., maximal blood lead above and below 10 μg/dL, and maximal blood lead above and below 7.5 μg/dL) (Figure 4). We then fit separate linear models to the data in each of these ranges and compared the blood lead coefficients for the concurrent blood lead index. The coefficient for the 103 children with maximal blood lead levels < 7.5 μg/dL was significantly greater than the coefficient for the 1,230 children with a maximal blood lead ≥7.5 μg/dL [linear β= −2.94 (95% CI, −5.16 to −0.71) vs. −0.16 (95% CI, −2.4 to −0.08); *p* = 0.015]. The coefficient for the 244 children who had a maximal blood lead < 10 μg/dL was not significantly greater than the coefficient for the 1,089 children who had a maximal blood lead ≥10 μg/dL [linear β= −0.80 (95% CI, −1.74 to −0.14) vs. β= −0.13 (95% CI, −2.3 to −0.03); *p* = 0.103].

To assess the model stability, we employed a random-effects model with sites assumed to be randomly selected from a larger set of populations. Results were similar to the preferred fixed-effects model, with the random-effects model producing a blood lead coefficient that was 3.7% lower (−2.6 vs. −2.7). As an additional measure of model stability, we fit seven identical log-linear models with each model omitting data from one of the sites. The range of coefficients leaving one site out at a time was −2.36 (Rochester) to −2.94 (Yugoslavia), or a percent change ranging from −2.6 to +8.9%. These analyses provide evidence of the stability of our final preferred fixed-effects model and indicate that the results of the pooled analysis did not depend on the data from any single study.

We also examined the relation of blood lead concentration to verbal and performance IQ scores, adjusting for the same covariates used in the full-scale IQ model. The coefficient for the log of blood lead related to performance IQ was similar to the coefficient for log of blood lead in the full-scale IQ model (β= −2.73 vs. −2.70), whereas the coefficient for log of blood lead related to verbal IQ was somewhat lower than the coefficient for the log of blood lead in the full-scale IQ model (β= −2.07 vs. −2.70). The difference between the coefficient for verbal and performance IQ was not statistically significant (*p* = 0.196).

We did not identify any significant interactions between the covariates and the log of concurrent blood lead. In the U.S. sites, race was not significantly associated with IQ after inclusion of the four covariates in the preferred model, nor did it alter the estimated relationship of blood lead concentration and IQ. In unadjusted analyses involving the 696 children who had cord blood lead levels, the log of cord blood lead concentration was significantly associated with child’s IQ (β= −1.69, SE = 0.60; *p* = 0.005). After adjusting for the log of concurrent blood concentration, the log of cord blood lead was no longer associated with children’s IQ scores (*p* = 0.21). In contrast, the log of concurrent blood lead was significantly associated with children’s IQ scores even with log cord blood lead concentration in the model (β= −1.73, SE = 0.74; *p* = 0.019). Finally, we identified and removed 65 potentially influential observations from the data and refit the model. The change in the coefficient for log of blood lead was 1.4%, from −2.70 to −2.74.

## Discussion

Before 1970, undue lead exposure was defined by a blood lead level of 60 μg/dL or higher—a level often associated with overt signs or symptoms of lead toxicity, such as abdominal colic, anemia, encephalopathy, and death. Since then, the blood lead concentration for defining undue lead exposure has been reduced: from 60 to 40 μg/dL in 1971, to 30 μg/dL in 1978, and to 25 μg/dL in 1985 (CDC 1991). In 1991, the CDC, and subsequently the WHO (1995), further reduced the blood lead value defining undue lead exposure to 10 μg/dL (CDC 1991). These ongoing reductions in the acceptable levels of children’s blood lead were motivated by evidence showing that blood lead concentrations as low as 10 μg/dL were associated with adverse effects, such as lower intelligence (CDC 1991; WHO 1995).

In this pooled analysis, we found evidence of lead-related intellectual deficits among children who had maximal blood lead levels < 7.5 μg/dL. Indeed, we found no evidence of a threshold. Other studies reported a similar finding, but questions about the relationship at lower levels remained because they involved smaller numbers of children with blood lead < 10 μg/dL or they did not adjust for important covariates (Canfield et al. 2003; Fulton et al. 1987; Lanphear et al. 2000; Schwartz 1994; Schwartz and Otto 1991; Walkowiak et al. 1998). In the pooled analysis, we estimated the blood lead–IQ relationship with data from the 5th to 95th percentile of the concurrent blood lead level at the time of IQ testing, which tends to underestimate the adverse effects of blood lead levels. For the entire pooled data set, the observed decline of 6.2 IQ points (95% CI, 3.8–8.6) for an increase in blood lead levels from < 1 to 10 μg/dL was comparable with the 7.4 IQ decrement for an increase in lifetime mean blood lead levels from < 1 to 10 μg/dL observed in the Rochester Longitudinal Study (Canfield et al. 2003).

Consistent with other studies (Bellinger and Needleman 2003; Canfield et al. 2003; Fulton et al. 1987; Lanphear et al. 2000; Schwartz 1994; Schwartz and Otto 1991; Walkowiak et al. 1998), the lead-associated IQ deficits observed in this pooled analysis were significantly greater at lower blood lead concentrations. In a meta-analysis, the observed decrement was greater in study cohorts in which children with blood lead levels < 15 μg/dL were more heavily represented (Schwartz 1994). In the Rochester Longitudinal Study, there was an estimated reduction of 7.4 IQ points for an increase in lifetime mean blood lead from 1 to 10 μg/dL (Canfield et al. 2003). In contrast, IQ scores declined 2.5 points for an increase in blood lead concentration from 10 to 30 μg/dL (Canfield et al. 2003). The larger sample size of this pooled analysis permitted us to show that the lead-associated intellectual decrement was significantly greater for children with a maximal blood lead of < 7.5 μg/dL than for those who had a maximal blood lead of ≥7.5 μg/dL. Although the difference in coefficients associated with the IQ decrement for children who had a maximal blood lead concentration < 10 μg/dL versus ≥10 μg/dL was not statistically significant, the results were consistent with the analysis using 7.5 μg/dL as a cut-point.

We found that concurrent blood lead levels or average lifetime estimates of lead exposure were generally stronger predictors of lead-associated intellectual deficits than was maximal measured (peak) or early childhood blood lead concentration. Although this finding conflicts with the widely held belief that 2-year (or peak) blood lead levels are the most salient measure of lead toxicity, there is increasing evidence that lifetime mean blood lead and concurrent blood lead levels are stronger predictors of IQ in older children (Baghurst et al. 1992; Canfield et al. 2003; Dietrich et al. 1993; Factor-Litvak et al. 1999). The stronger effects of concurrent and lifetime measures of lead exposure may be due to chronicity of exposure (Bellinger and Dietrich 1994). Alternatively, the weaker association with blood lead measured during early childhood may be due to exposure misclassification from the greater within-child variability of blood lead in younger children. Nevertheless, because blood lead concentrations taken in early childhood track closely with subsequent blood lead levels (Baghurst et al. 1992; Canfield et al. 2003; Dietrich et al. 1993), we cannot entirely resolve the question of whether children are more vulnerable to lead exposure during the first 2 years of life. Still, young children do ingest more lead during the first 2 years of life and may absorb it more efficiently than do older children and adults (Clark et al. 1985; Lanphear et al. 2002; Ziegler et al. 1978). Thus, efforts to prevent lead exposure must occur before pregnancy or a child’s birth.

The specific mechanisms for lead-induced intellectual deficits have not been fully elucidated. There are several plausible mechanisms for the greater lead-associated intellectual deficits observed at blood lead levels < 10 μg/dL (Lidsky and Schneider 2003; Markovac and Goldstein 1988; Schneider et al. 2003), but it is not yet possible to link any particular mechanism with the deficits observed in this pooled analysis. Nevertheless, efforts can be taken to reduce environmental lead exposure without full elucidation of the underlying mechanism (Wynder 1994).

The observational design of this study limits our ability to draw causal inferences. Instead, we must rely on the consistency of findings from numerous epidemiologic and experimental studies in rodents and nonhuman primates, including evidence that environmental lead exposure is associated with intellectual deficits at blood lead levels < 10 μg/dL. There are potential limitations of the tools we used to measure important covariates. The HOME Inventory was not conducted at the same age for children in all of the sites, and the HOME Inventory and IQ tests have not been validated in all cultural or ethnic communities. Nonetheless, because these covariates were standardized and adjusted for study site, these problems do not pose any limitations to the interpretation of the pooled analysis results. There are other predictors of neurodevelopmental outcomes that we did not examine in this pooled analysis, such as maternal depression. The omission of unmeasured variables may produce residual confounding (Pocock et al. 1994). Still, in studies that did examine other relevant covariates, such as breast-feeding and iron status, the estimated effect of lead was not altered appreciably (Canfield et al. 2003; Needleman et al. 1990; Tong and Lu 2000). Finally, each of the cohorts has unique limitations that raise questions about the validity and generalizability of their findings. Nevertheless, the results of these analyses indicate that the results are robust and not dependent on the data from any one site.

The impact of low-level environmental lead exposure on the health of the public is substantial. This pooled analysis focused on intellectual deficits, but environmental lead exposure has been linked with an increased risk for numerous conditions and diseases that are prevalent in industrialized society, such as reading problems, school failure, delinquent behavior, hearing loss, tooth decay, spontaneous abortions, renal disease, and cardiovascular disease (Borja-Aburto et al. 1999; Dietrich et al. 2001; Factor-Litvak et al. 1999; Lin et al. 2003; Moss et al. 1999; Nash et al. 2003; Needleman et al. 2002; Schwartz and Otto 1991). Although only a few studies have examined the association of these conditions or diseases among individuals with blood lead levels < 10 μg/dL (Borja-Aburto et al. 1999; Lanphear et al. 2000; Moss et al. 1999; Schwartz and Otto 1991), the evidence is growing.

In conclusion, the results of this pooled analysis underscore the increasing importance of primary prevention as the consequences of lower blood lead concentrations are recognized. Although blood lead concentrations < 10 μg/dL in children are often considered “normal,” contemporary blood lead levels in children are considerably higher than those found in pre-industrial humans (Patterson et al. 1991). Moreover, existing data indicate that there is no evidence of a threshold for the adverse consequences of lead exposure. Collectively, these data provide sufficient evidence to eliminate childhood lead exposure by banning all nonessential uses of lead and further reducing the allowable levels of lead in air emissions, house dust, soil, water, and consumer products (Lanphear 1998; Rosen and Mushak 2001).

## Figures and Tables

**Table 1:** Characteristics of the children and of their mothers in the pooled analysis (*n* = 1,333).

Characteristic	Value
Child characteristics	
Female*^a^*	669 (50.2)
Birth weight*^b^* (g)	3,286 ± 503
Gestation at delivery*^b^* (weeks)	39.6 ± 1.9
Birth order*^c^*	2.0 (1–5)
Blood lead concentration*^c^*	
Concurrent	9.7 (2.5–33.2)
Peak	18.0 (6.2–47.0)
Early childhood	12.7 (4.0–34.5)
Lifetime average	12.4 (4.1–34.8)
Peak blood lead concentration < 10 μg/dL*^a^*	244 (18.3)
Peak blood lead concentration < 7.5 μg/dL*^a^*	103 (7.7)
IQ*^b^*	93.2 ± 19.2
Age at IQ testing*^b^* (years)	6.9 ± 1.2
Maternal characteristics	
Age at delivery*^b^* (years)	25.4 ± 5.4
Maternal IQ*^b^*	88.2 ± 18.5
Education at delivery*^b^* (grade)	11.1 ± 2.8
HOME score*^b^*	37.0 ± 8.4
Married*^a^*	896 (67.3)
Smoked during pregnancy*^a^*	453 (34.1)
Alcohol use during pregnancy*^a^*	278 (21.2)

HOME score was standardized to preschool test. Early childhood blood lead concentration was defined as the mean of 6- to 24-month blood lead tests. Lifetime average blood lead concentration was defined as the mean of blood lead tests taken from 6 months through the concurrent blood lead test.

a No. (%).

b Mean ± SD.

c Median (5th–95th percentiles).

**Table 2:** Characteristics of 1,333 children and their mothers in seven cohort studies of environmental lead exposure and IQ.

Characteristic	Boston (*n* = 116)	Cincinnati (*n* = 221)	Cleveland (*n* = 160)	Mexico (*n* = 99)	Port Pirie (*n* = 324)	Rochester (*n* = 182)	Yugoslavia (*n* = 231)
Percent female*^a^*	60 (51.7)	108 (48.9)	73 (45.6)	50 (50.5)	174 (53.7)	89 (48.9)	115 (49.8)
Birth weight*^b^* (g)	3,412 ± 510	3,144 ± 457	3,199 ± 498	3,254 ± 432	3,393 ± 502	3,226 ± 506	3,328 ± 526
Gestation at delivery*^b^* (weeks)	40.0 ± 1.8	39.6 ± 1.7	39.6 ± 1.2	40.2 ± 1.1	39.9 ± 1.7	39.1 ± 1.8	39.3 ± 2.9
Birth order*^b^*	1.6 ± 1.0	2.6 ± 1.4	2.2 ± 1.1	1.8 ± 0.9	2.0 ± 1.1	2.4 ± 1.4	2.6 ± 1.7
IQ test	WISC-R	WISC-R	WPPSI	WISC-S	WISC-R	WPPSI	WISC-III
IQ score*^b^*	116.0 ± 14.2	87.0 ± 11.4	86.7 ± 16.2	107.8 ± 11.0	106.0 ± 13.7	84.9 ± 14.4	74.2 ± 13.3
Age at IQ testing (years)	10	7	4.8	7	7	6	7
Blood lead concentrations*^c^*							
Concurrent blood lead	5.4 (0.8–12.7)	7.5 (3.5–20.0)	14.2 (7.0–28.5)	7.0 (3.0–16.5)	13.0 (6.0–24.0)	4.0 (1.5–12.0)	15.9 (4.7–47.8)
Peak blood lead	12.0 (5.4–27.0)	17.9 (9.0–38.0)	18.0 (9.0–34.0)	15.0 (6.0–40.0)	27.0 (15.0–46.0)	9.0 (3.5–23.3)	23.8 (7.6–61.5)
Early childhood	8.1 (3.3–18.0)	12.0 (6.6–26.6)	13.4 (7.9–24.8)	11.4 (4.3–26.8)	20.5 (11.0–33.3)	5.8 (2.4–13.1)	14.1 (4.3–44.0)
Lifetime mean	7.6 (3.6–15.2)	11.7 (5.8–24.9)	14.5 (8.1–25.3)	10.6 (4.5–21.3)	18.6 (10.8–30.2)	5.5 (2.4–12.8)	15.8 (5.6–49.3)
Peak blood lead < 10 μg/dL*^a^*	41 (35.3)	23 (10.4)	11 (6.9)	20 (20.2)	0 (0.0)	103 (56.6)	46 (19.9)
Peak blood lead < 7.5 μg/dL*^a^*	13 (11.2)	1 (0.4)	1 (0.6)	8 (8.1)	0 (0.0)	69 (37.9)	11 (4.8)
Maternal characteristics							
Age at delivery (years)*^b^*	30.5 ± 4.2	22.7 ± 4.3	22.2 ± 3.8	27.1 ± 5.9	26.0 ± 4.2	24.8 ± 6.6	26.6 ± 5.1
Race (nonwhite)*^a^*	5 (4.3)	197 (89.1)	69 (43.1)	NA	NA	130 (71.4)	NA
Maternal IQ*^b^*	124.2 ± 16.2	75.2 ± 9.4	73.4 ± 13.2	93.4 ± 11.9	94.4 ± 11.0	81.1 ± 12.6	87.3 ± 14.8
Education at delivery (grade)*^b^*	15.2 ± 2.0	11.2 ± 1.4	10.6 ± 1.6	11.4 ± 3.5	10.6 ± 1.0	12.2 ± 2.0	8.8 ± 3.9
HOME score*^b^*	50.5 ± 3.5	32.7 ± 6.2	38.1 ± 6.7	36.8 ± 6.7	42.3 ± 4.6	31.9 ± 6.3	30.4 ± 6.8
Married*^a^*	107 (92.2)	30 (13.6)	82 (51.2)	88 (88.9)	298 (92.0)	60 (33.2)	231 (100)
Tobacco use during pregnancy*^a^*	29 (25.0)	111 (50.2)	128 (80.0)	6 (6.1)	79 (24.6)	41 (22.6)	59 (25.5)
Alcohol use during pregnancy*^a^*	61 (52.6)	31 (14.0)	75 (46.9)	6 (6.1)	82 (25.3)	9 (5.5)	14 (6.1)

NA, Not applicable. HOME score was standardized to preschool scale. Concurrent blood lead tests taken at 5 years of age were used as the concurrent blood lead test for the Boston cohort, and the IQ test was done at 10 years. Test scores of children in the Yugoslavia cohort are low because of adjustments in adapting tests where no standardization existed; rather than deriving appropriate analogues, some culturally driven items were removed, resulting in lower scores.

a No. (%).

b Mean ± SD.

c Geometric mean (5th–95th percentiles).

**Table 3:** Concurrent blood lead concentration and mean IQ scores by characteristics of children and their mothers (*n* = 1,333).

Covariate	No.	Median concurrent blood lead (μg/dL) (5th–95th percentiles)	IQ ± SD
Child			
Female	669	9.0 (2.4–31.4)	93.8 ± 18.3
Male	664	9.9 (2.6–35.7)	92.5 ± 20.0
Birth weight (g)			
< 3,000	359	10.0 (2.2–28.7)	88.6 ± 18.0
3,000 to < 3,500	519	9.9 (2.4–34.2)	93.6 ± 19.3
≥3,500	455	9.1 (2.8–34.7)	96.3 ± 19.3
Gestation at delivery (weeks)			
< 38	144	8.9 (3.1–37.9)	83.5 ± 18.6
38 to < 42	1,071	9.8 (2.5–33.2)	94.1 ± 18.6
≥42	115	10.0 (3.2–24.8)	96.3 ± 22.1
Birth order			
1	479	9.0 (2.1–32.6)	96.7 ± 18.9
2	407	10.0 (2.6–31.4)	93.6 ± 19.2
≥3	446	10.0 (3.0–36.9)	89.0 ± 18.7
Maternal			
Race (only U.S. cohorts)			
White	278	7.9 (1.3–22.0)	100.6 ± 20.1
Nonwhite	401	7.1 (2.8–21.5)	84.9 ± 12.8
Age at delivery (years)			
< 25	650	10.5 (3.0–32.0)	89.6 ± 17.2
≥25	683	9.0 (2.1–34.7)	96.5 ± 20.3
Maternal IQ			
< 85	618	10.0 (2.9–32.0)	83.3 ± 15.0
≥85	715	9.0 (2.1–34.3)	101.6 ± 18.3
Education at delivery (grade)			
< 12	710	12.0 (4.1–35.5)	90.4 ± 18.8
12	397	8.7 (2.4–34.3)	91.1 ± 17.7
≥12	226	5.5 (1.1–15.2)	105.5 ± 18.0
HOME score			
< 30	276	9.4 (3.0–43.0)	77.9 ± 14.9
30 to < 40	561	10.0 (2.8–32.2)	88.3 ± 15.4
≥40	496	9.5 (2.0–22.0)	107.0 ± 15.8
Married			
Yes	896	10.0 (2.7–37.5)	96.2 ± 20.5
No	436	8.1 (2.4–22.0)	87.0 ± 14.3
Prenatal smoking			
Yes	453	11.5 (3.2–33.2)	89.5 ± 17.2
No	876	8.7 (2.2–33.6)	94.9 ± 19.9
Prenatal alcohol ingestion			
Yes	278	10.1 (2.2–25.0)	99.3 ± 19.4
No	1,035	9.5 (2.7–34.3)	91.7 ± 18.8

**Table 4:** Mean unadjusted and adjusted*^a^* changes in full-scale IQ score associated with an increase in blood lead concentration (log scale), from the 5th to 95th percentile of the concurrent blood lead level at the time of IQ testing.

Blood lead variable	Unadjusted estimates [β(95% CI)]	Adjusted estimates [β(95% CI)]	Blood lead concentration (5th to 95th percentile, μg/dL)	IQ deficits [5th to 95th percentile (95% CI)]
Early childhood	−3.57 (−4.86 to −2.28)	−2.04 (−3.27 to −0.81)	4.1–34.8	4.4 (1.7–7.0)
Peak	−4.85 (−5.16 to −3.54)	−2.85 (−4.10 to −1.60)	4.0–34.5	6.1 (3.4–8.8)
Lifetime average	−5.36 (−6.69 to −4.03)	−3.04 (−4.33 to −1.75)	6.1–47.0	6.2 (3.6–8.8)
Concurrent	−4.66 (−5.72 to −3.60)	−2.70 (−3.74 to −1.66)	2.4–33.1	7.1 (4.4–9.8)

a Adjusted for site, HOME score, birth weight, maternal IQ, and maternal education. The addition of child’s sex, tobacco exposure during pregnancy, alcohol use during pregnancy, maternal age at delivery, marital status, and birth order did not alter the estimate, and these were not included in the model. The estimates for the covariates in the concurrent blood lead model were HOME score (β= 4.23, SE = 0.54), birth weight/100 g (β= 1.53, SE = 0.35), maternal IQ (β= 4.77, SE = 0.57), and maternal education (β= 1.12, SE = 0.46).
